# Supplementary figures and images for: Single-Cell and Bulk RNA Sequencing Reveal Malignant Epithelial Cell Heterogeneity and Prognosis Signatures in Gastric Carcinoma
Source: Cells. 2022 Aug 17;11(16):2550. doi: 10.3390/cells11162550 (PMC9407012; doi:10.3390/cells11162550)

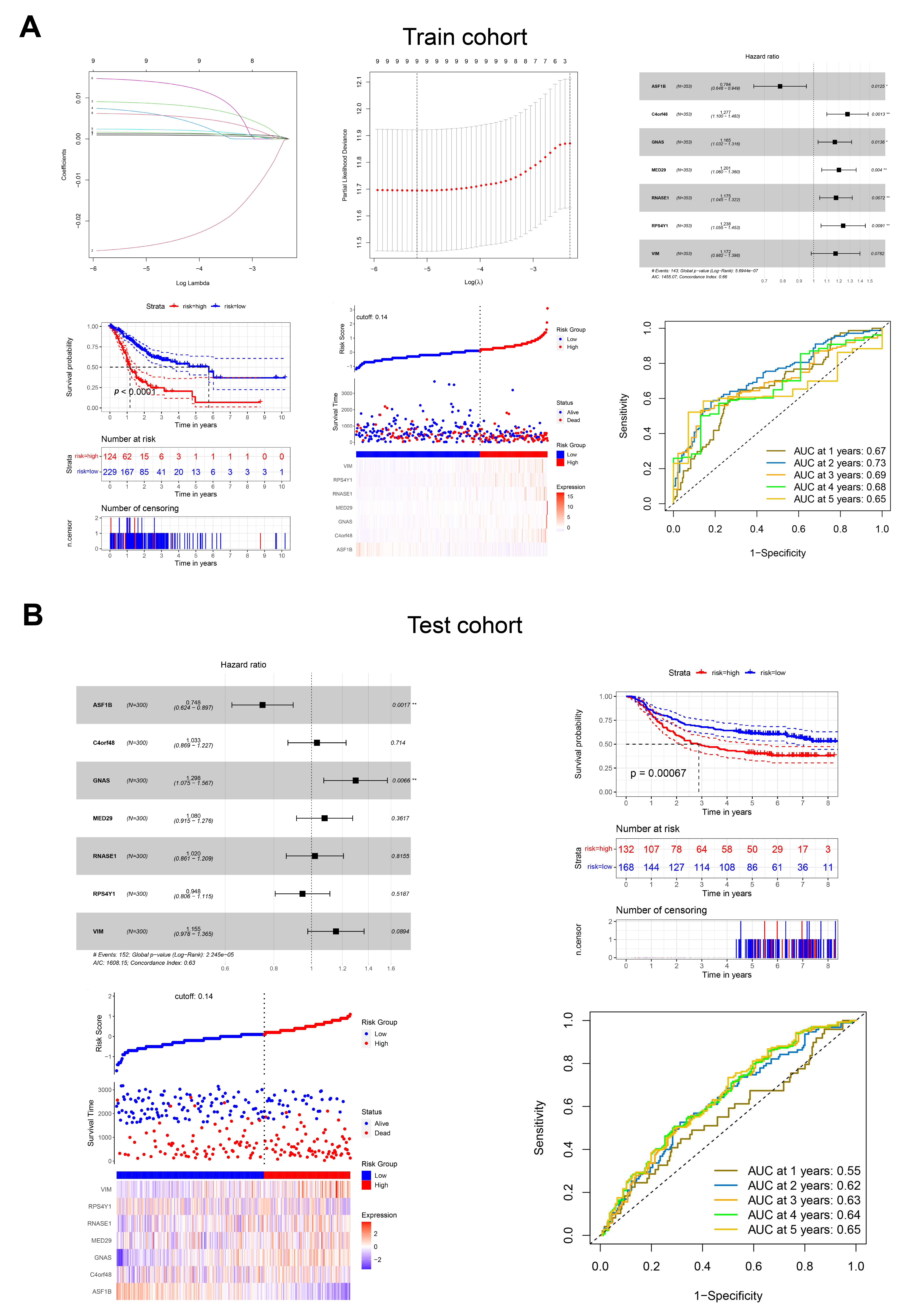

Supplement: Supplementary file 1 [file cells-11-02550-s001.zip › Supplementary Figure S1.tif]

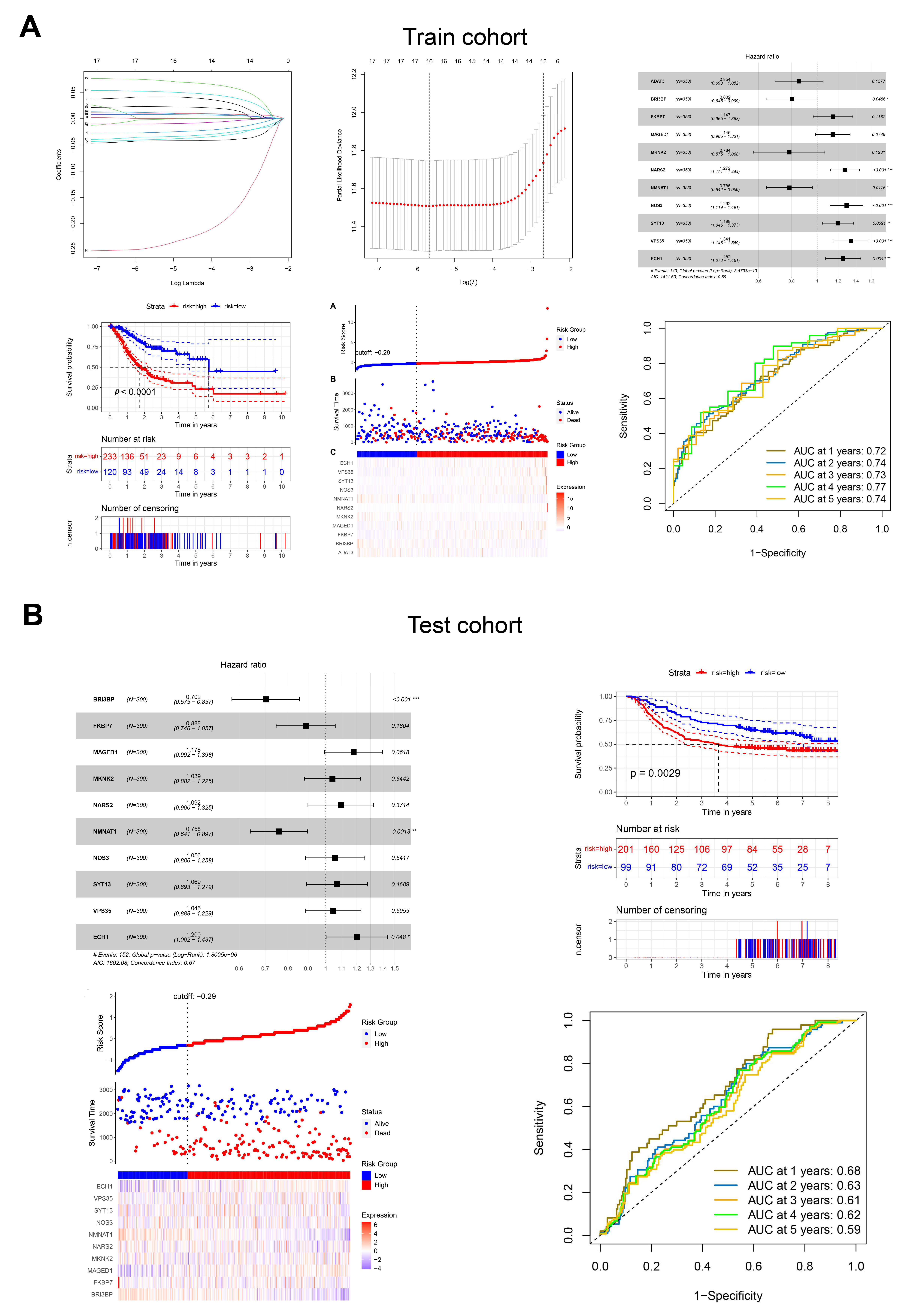

Supplement: Supplementary file 1 [file cells-11-02550-s001.zip › Supplementary Figure S2.tif]

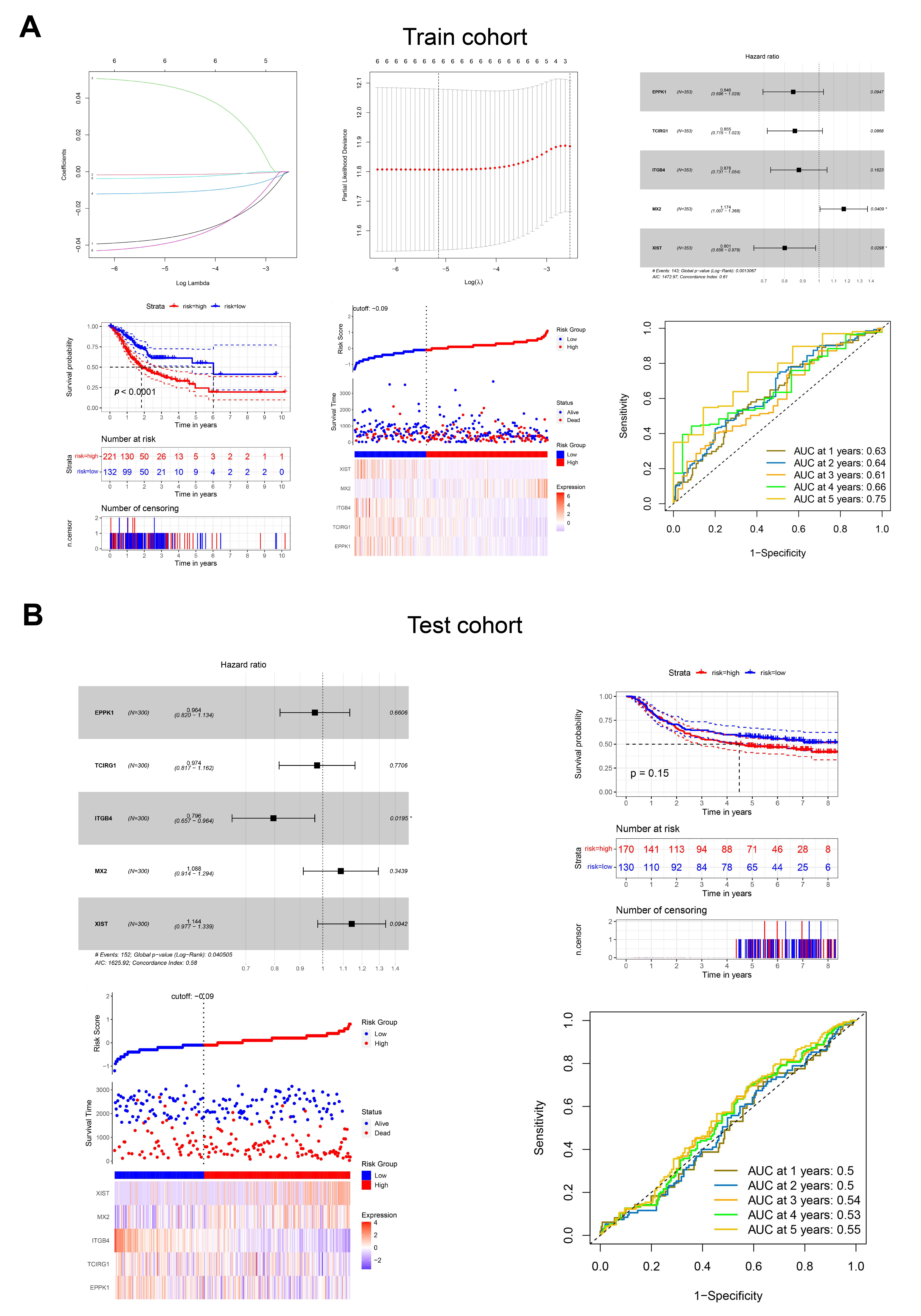

Supplement: Supplementary file 1 [file cells-11-02550-s001.zip › Supplementary Figure S3.tif]
